# Supplementary material for: Heat adaptation of phage T7 under an extended genetic code
Source: Virus Evol. 2021 Dec 1;7(2):veab100. doi: 10.1093/ve/veab100 (PMC8923235; doi:10.1093/ve/veab100)
Supplement: veab100_Supp [file veab100_supp.zip › Sequencing_Analysis.zip!/Sequencing_Analysis/A3/evidence/SNP_8.html]

BRESEQ :: Evidence


| Predicted mutation | | | | | | |
| --- | --- | --- | --- | --- | --- | --- |
| evidence | seq id | position | mutation | annotation | gene | description |
| RA | NC\_001604.1 | 24,475 | A→G | intergenic (–/–) | – / – | –/– |

| Read alignment evidence... | | | | | | | | | | | |
| --- | --- | --- | --- | --- | --- | --- | --- | --- | --- | --- | --- |
|  | seq id | position | | ref | new | freq | score (cons/poly) | reads | annotation | genes | product |
| \* | NC\_001604.1 | 24,475 | 0 | A | G | 99.7% | 5486.8 / NA | 1831 | intergenic (–/–) | *–/–* | –/– |
| Reads supporting (aligned to +/- strand):  **ref** base A (2/3);  **major** base G (957/861);  **minor** base T (3/3);  **total** (964/867) | | | | | | | | | | | |
| Fisher's exact test for biased strand distribution *p*-value = 1.00e+00 | | | | | | | | | | | |
| Kolmogorov-Smirnov test that lower quality scores support variant *p*-value = 1.00e+00 | | | | | | | | | | | |

Only 100 of 1838 total aligned reads displayed.  

`GGGGCCTCTAAACGGGTCTTGAGGGGTTTTTTGCTGAAAGGAGGAACTATATGCGCTCATACGATATGAACGTTGAGACTGCCGCTGAGTTATCAGCTGTGAACGACATTCTGGCGTCTATCGGTGAACCTCCGGTATCAACGCTGGAAGGTGACGCTAACGCAGATGCAGCGAACGCTCGGCGTATTCTCAACAAGATTAACCGACAGATTCAATCTCGTGGATGGACGTTCAACATTGAGGAAGGCATAACGCTACTACCTGATGTTTACTCCAACCTGATTGTATACAGTGACGACTATTTATCCCTAATGTCTACTTCCGGTCAATCCATCTACGTTAACCGAGGTGGCTATGTGTATGACCGAACGAGTCAATCAGACCGCTTTGACTCTGGTATTACTGTGAACATTATTCGTCTCCGCGACTACGATGAGATGCCTGAGTGCTTCCGTTACTGGATTGTCACCAAGGCTTCCCGTCAGTTCAACAACCGATTCTTTGGGGCACCGGAAGTAGAGGGTGTACTCCAAGAAGAGGAAGATGAGGCTAGACGTCTCTGCATGGAGTATGAGATGGACTACGGTGGGTACAA  >  NC_001604.1/24178‑24772`  

                                                                                                                                                                                                                                                                                                         `|`                                                                                                                                                                                                                                                                                                           
   `ataagagacagggTCTTGAGGGGTTTTTTGCTGAAAGGATGAACTATATGCGCTCATGCGATATGAACGTTGAGACTGCCGCTGAGTTATCAGCTGTGAACGACATTCTGGCGTCTATCGGTGAACCTCCGGTATCAACGCTGGAAGGTGACGCTAACGCAGATGCAGCGAACGCTCGGCGTATTCTCAACAAGATTAACCGACAGATTCAATCTCGTGGATGGACGTTCAACATTGAGGAAGGCATAACGCTACTACCTGATGTTTACTCCAACCTGATTGTATACAGTGACGGCTAttt                                                                                                                                                                                                                                                                                                     <  1:215780/291‑1 (MQ=255)`  
    `ccTCTAAACGGGTCTTGAGGGGTTTTTTGCTGAAAGGAGGAACTATATGCGCTCATGCGATATGAACGTTGAGACTGCCGCTGAGTTATCAGCTGTGAACGACATTCTGGCGTCTATCGGTGAACCTCCGGTATCAACGCTGGAAGGTGACGCTAACGCAGATGCAGCGAACGCTCGGCGTATTCTCAACAAGATTAACCGACAGATTCAATCTCGTGGATGGACGTTCAACATTGAGGAAGGCATAACGCTCCTACATGATGTTTACTCCAACCTGATTGTATACAGTGACGGCTAtttt                                                                                                                                                                                                                                                                                                    >  1:305142/1‑300 (MQ=255)`  
    `ccTCTAAACGGGTCTTGAGGGGTTTTTTGCTGAAAGGAGGAACTATATGCGCTCATGCGATATGAACGTTGAGACTGCCGCTGAGTTATCAGCTGTGAACGACATTCTGGCGTCTAACGGTGAACCTCCGGTATCAACGCTGGAAGGTGACGCTAACGCAGATGCAGCGAACGCTCGGCGTATTCTCAACAAGATTAACCGACAGATTCAATCTCGTGGATGGACGTTCAACATTGAGGAAGGCATAACGCTACTACCTGATGTTTACTACAACCTGATTGTATCCAGTGACGGCTATTTa                                                                                                                                                                                                                                                                                                    >  1:315914/1‑301 (MQ=255)`  
       `ctAAACGGGTCTTGAGGGGTTTTTTGCTGAAAGGAGGAACTATATGCGCTCATGCGATATGAACGTTGAGACTGCCGCTGAGTTATCAGCTGTGAAAGACATTCTGGCGTCTATCGGTGAACCTCCGGTATCAACGCTGGAAGGTGACGCTAACGCAGATGCAACGAACGCTCGGCGTATTCTCAACAAGATTAACCGACAGATTCAATATCGTGGATGGACGTTCAACATTGAGGAAGGCATAACGCTACTACCTGATGTTTACTCCAACCTGATTGTATACAGTGACGGCTATTTATcc                                                                                                                                                                                                                                                                                                 >  1:108391/1‑301 (MQ=255)`  
        `tAAACTGGTCTTGAGGGGTTTTTTGCTGAAAGGAGGAACTATAGGCGCTCATGCGATATGAACGTTGAGACTGCCGCTGAGTTATCAGCTGTGAACGAAATTCTGGCGTCTATCGGTGAACCTCCGGTATCAACGCTGGAAGGTGACGCTAACGCAGATGCAGCGAACGCTCGGCGTATTCTCAACAAGATTAACCGACAGATTCAATCTCGTGGATGGACGTTCAACATTGAGGAAGGCATAACGCTACTACCTGATGTTTACTCCAACCTGATTGTATACAGTGACGGCTATTTATccc                                                                                                                                                                                                                                                                                                <  1:111560/301‑1 (MQ=255)`  
        `aaaaCGGGTCTTGAGGGGTTTTTTGCTGAAAGGAGGAACTATATGCGCTCATGCGATATGAACGTTGAGACTGCCGCTGAGTTATCAGCTGTGAACGACATTCTGGCGTCTATCGGTGAACCTCTGGTATCAACGCTGGAAGGTGACGCTAACGCAGATGCAGCGAACGCTCGGCGTATTCTCAACAAGATTAACCGACAGATTCAATCTCGTGGATGGACGTTCAACATTGAGGAAGGCATAACGCTACTACCTGATGTTTACTCCAACCTGATTGTATACAGTGACGGCTATTTATccc                                                                                                                                                                                                                                                                                                <  1:235353/300‑1 (MQ=255)`  
               `ggtgggctcggagatgtgtataagagacagaCTATATGCGCTCATGCGATATGAACGTTGAGACTGCCGCTGAGTTATCAGCTGTGAACGACATTCTGGCGTCTATCGGTGAACCTCCGGTATCAACGCTGGAAGGTGACGCTAACGCAGATGCAGCGAACGCTCGGCGTATTCTCAACAAGATTAACCGACAGATTCAATCTCGTGGATGGACGTTCAACATTGAGGAAGGCATAACGCTACTACCTGATGTTTACTCCAACCTGATTGTATACAGTGACGGCTATTTATCCCTAATGTc                                                                                                                                                                                                                                                                                         <  1:62527/271‑1 (MQ=255)`  
               `gTCTTGAGGGGTTTTTTGCGGAAAAGAGGAAATATATGCGCTCATGCGATATGAACGTTGAGAAGGCCGGTGAGTTATCAGCTGTGAACGACATTCTGGCGTCTATCGGTGAACCTCCGGTATCAACGCTGGAAGGTGACGCTAACGCAGATGCAGCGAACGCTCGGCGTATTCTCAACAAGATTAACCGACAGATTCAATCTCGTGGATGGACGTTCAACATTGAGGAAGGCATAACGCTACTACCTGATGTTTACTCCAACCTGATTGTATACAGTGACGGCTATTTATCCCTAATGTc                                                                                                                                                                                                                                                                                         <  1:138757/301‑1 (MQ=255)`  
                  `ttGAGGGGTTTTTTGCTGAAAGGAGGAACTATATGCGCTCATGCGATATGAACGTTGAGACTGCCGCTGAGTTATCAGCTGTGAACGACATTCTGGCGTCTATCGGTGAACCTCCGGTATCAACGCTGGAAGGTGACGCTAACGCAGATGCAGCGAACGCTCGGCGTATTCTCAACAAGATTAACCGACAGATTCAATCTCGTGGATGGACGTTAAACATTGAGGAAGGCATAACGCTACTACCTGATGTTTACTCCAACCTGATTGTATACAGTGACGGCTATTTATCCCTAATGTCTAc                                                                                                                                                                                                                                                                                      <  1:133507/301‑1 (MQ=255)`  
                     `gggggTTTTTTGCTTAAAGGAGTAACTATAGGCGCTCATGAGATATGAACGTTGAGACTGCCGCTGAGTTATCAGCTGTGAACGACATTCTGGCGTCTATCGGTGAACCTCCGGTATCAACGCTGGAAGGTGACGCTAACGCAGATGCAGCGAACGCTCGGCGTATTCTCAACAAGATTAACCGACAGATTCAATCTCGTGGATGGACGTTCAACATTGAGGAAGGCATAACGCTACTACCTGATGTTTACTCCAACCTGATTGTATACAGTGACGGCTATTTATCCCTAATGTCTACTTc                                                                                                                                                                                                                                                                                   <  1:299188/300‑1 (MQ=255)`  
                     `agatgtgtataagagacaggaggAACTATATGCGCTCATGCGATATGAACGTTGAGACTGCCGCTGAGTTATCAGCTGTGAACGACATTCTGGCGTCTATCGGTGAACCTCCGGTATCAACGCTGGAAGGTGACGCTAACGCAGATGCAGCGAACGCTCGGCGTATTCTCAACAAGATTAACCGACAGATTCAATCTCGTGGATGGACGTTCAACATTGAGGAAGGCATAACGCTACTACCTGATGTTTACTCCAACCTGATTGTATACAGTGACGGCTGTTTATCCCTAATGTCTACTTc                                                                                                                                                                                                                                                                                   <  1:70141/284‑1 (MQ=255)`  
                     `aGGGGGTTTTTGCTGAAAGGAGGAACTATATGCGCTCATGCGATATGAACGTTGAGACTGCCGCTGAGTTATCAGCTGTGAACGACATTCTGGCGTCTATCGGTGAACCTCCGGTATCAACGCTGGAAGGTGACGCTAACGCAGATGCAGCGAACGCTCGGCGTATTCTCAACAAGATTAACCGACAGATTCAATCTCGTGGATGGACGTTCAACATTGAGGAAGGCATAACGCTACTACCTGATGTTTACTCCAACCTGATTGTATACAGTGACGGCTATTTATCCCTAATGTCTACTTc                                                                                                                                                                                                                                                                                   <  1:39210/301‑1 (MQ=255)`  
                      `ggctcggagatgggtataagagacagatagGCGATCATGCGATATGAACGGTGAGACTGCCGCTGAGTTATCAGCTGTGAACGACATTCTGGCGTCTATCGGTGAACCTCCGGTATCAACGCTGGAAGGTGACGCTAACGCAGATGCAGCGAACGCTCGGCGTATTCTCAACAAGATTAACCGACAGATTCAATCTCGTGGATGGACGTTCAACATTGAGGAAGGCATAACGCTACTACCTGATGTTTACTCCAACCTGATTGTATACAGTGACGGCTATTTATCCCTAATGTCTACTTcc                                                                                                                                                                                                                                                                                  <  1:136050/275‑1 (MQ=255)`  
                         `gTTTTTTGCTGAAAGGAGGAACTATATGCGCTCATGCGATATGAACGTTGAGACTGCCGCTGAGTTATCAGCTGTGAACGACATTCTGGCGTCTATCGGTGAACCTCCGGTATCAACGCTGGAAGGTGACGCTAACGCAGATGCAGCGAACGCTCGGCGTATTCTCAACAAGATTAACCGACAGATTCAATCTCGTGGATGGACGTTCAACATTGAGGAAGGCATAACGCTACTACCTGATGTTTACTCCAACCTGATTGTATACAGTGACGGCTATTTATcccctgtctcttatacacat                                                                                                                                                                                                                                                                               >  1:156955/1‑284 (MQ=255)`  
                                    `ggagatttgtataagagacaccATGCGATATGAACGTTGAGACTGCCGCTGAGTTATCAGCTGTGAACGACATTCTGGCGTCTATCGGTGAACCTCCGGTATCAACGCTGGAAGGTGACGCTAACGCAGATGCAGCGAACGCTCGGCGTATTCTCAACAAGATTAACCGACAGATTCAATCTCGTGGATGGACGTTCAACATTGAGGAAGGCATAACGCTACTACCTGATGTTTACTCCAACCTGATTGTATACAGTGACGGCTATTTATACCTAATGTCTACTTCCGGTCAATCCATCTa                                                                                                                                                                                                                                                                    <  1:57125/280‑1 (MQ=255)`  
                                       `ggaggaACTATATGCGCTCATGCGATATGAACGTTGAGACTGCCGCTGAGTTATCAGCTGTGAACGACATTCTGGCGTCTATCGGTGAACCTCCGGTATCAACGCTGGAAGGTGACGCTAACGCAGATGCAGCGAACGCTCGGCGTATTCTCAACAAGATTAACCGACAGATTCAATCTCGTGGATGGACGTTCAACATTGAGGAAGGCATAACGCTACTACCTGATGTTTACTCCAACCTGATTGTATACAGTGACGGCTATTTATCCCTAATGTCTACTTCCGGTCAATCCATCTACGt                                                                                                                                                                                                                                                                 >  1:170337/1‑301 (MQ=255)`  
                                           `gaACTATATGCGCTCATGCGATATGAACGTTGATACTGCCGCTGAGTTATCAGCTGTGAACGACATTCTGGCGTCTATCGGTGAACCTCCGGTATCAACGCTGGAAGGTGACGCTAACGCAGATGCAGCGAACGCTCGGCGTATTCTCAACAAGATTAACCGACAGATTCAATCTCGTGGATGGACGTTCAACATTGAGGAAGGCATAACGCTACTACCTGATGTTTACTCCAACCTGATTGTATACAGTGACGGCTATTTATCCCTAATGTCTAcctgtctcttatacacatctccgagc                                                                                                                                                                                                                                                             >  1:203923/1‑276 (MQ=255)`  
                                              `cTATATGCGCTCATGCGATATGAACGTTGAGACTGCCGCTGAGTTATCAGCTGTGAACTACATTCTGGCGTCTATCGGTGAACCTCCGGTATCAACGCTGGAAGGTGATGCTAACGCAGATGCAGCGAACGCTCGGCGTATTCTCAACAAGATTAACCGACAGATTCAATCTCGTGGATGGACGTTCAACATTGAGGAAGGCATAACGCTACTACCTTATGTTTACTCCAACCTGATTGTATACAGTGACGGCTATTTATCCCTAATGTCTACTTCCGGTCAATCCATCTACGTTAACCGa                                                                                                                                                                                                                                                          >  1:45181/1‑301 (MQ=255)`  
                                                `atatGTGCTCATGCGATATGAACGTTGAGACTGCCGCTGAGTTATCAGCTGTGAACGACATTCTGGCGTCTATCGGTGAACCTCCGGTATCAACGCTGGAAGGTGACGCTAACGCAGATGCAGCGAACGCTCGGCGTATTCTCAACAAGATTAACCGACAGATTCAATCTCGTGGATGGACGTTCAACATTGAGGAAGGCATAACGCTACTACCTGATGTTTACTCCAACCTGATTGTATACAGTGACGGCTATTTATCCCTAATGTCTACTTCCGGTCAATCCATCTACGTTAACCGAgg                                                                                                                                                                                                                                                        >  1:64252/1‑301 (MQ=255)`  
                                                `atatGCGCTCATGCGATATGAACGTTGAGACTGCCGCTGAGTTATCAGCTGTGAACGACATTCTGGCGTCTATCGGTGAACCTCCGGTATCAACGCTGGAAGGTGACGCTAACGCAGATGCAGCGAACGCTCGGCGTATTCTCAACAAGATTAACCGACAGATTCAATCTCGTGGATGGACGTTCAACATTGAGGAAGGCATAACGCTACTACCTGATGTTTACTCCAACCTGATTGTATACAGTGACGGCTATTTATCCCTAATGTCTACTTCCGGTCAATCCATCTACGtctgtctctt                                                                                                                                                                                                                                                        >  1:174864/1‑292 (MQ=255)`  
                                                         `cATGCGATATGAACGTTGAGACTGCCGCTGAGTTATCAGCTGTGAACGACATTCTGGCGTCTATCGGTGAACCTCCGGTATCAACGCTGGAAGGTGACGCTAACGCAGATGCAGCGAACGCTCGGCGTATTCTCAACAAGATTAACCGACAGATTCAATCTCGTGGATGGACGTTCAACATTGAGGAAGGCATAACGCTACTACCTGATGTTTACTCCAACCTGATTGTATACAGTGACGGCTATTTATCCCTAATGTCTACTTCCGGTCAATCCATCTACGTTAACCGAGGTGGCCAtgt                                                                                                                                                                                                                                               >  1:260002/1‑301 (MQ=255)`  
                                                          `ggggctcggagatgtgtataagagacaggAGTTATCAGCTGTGAACGACATTCTGGCGTCTATCGGTGAACCTCCGGTATCAACGCTGGAAGGTGACGCTAACGCAGATGCAGCGAACGCTCGGCGTATTCTCAACAAGATTAACTGACAGATTCAATCTCGTGGATGGACGTTCAACATTGAGGAAGGCATAACGCTACTACCTGATGTTTACTCCAACCTGATTGTATACAGTGACGGATATTTATCCCTAATGTCTACTTCCGGTCAATCCATCTACGTTAACCGAGGTGGCTAtgtg                                                                                                                                                                                                                                              <  1:123004/273‑1 (MQ=255)`  
                                                          `atgcGATATGAACGTTGAGACTGCCGCTGAGTTATCAGCTGTGAACGACATTCTGGAGTCTATCGGTGAACCTCCGGTATCAACGCTGGAAGGTGACGCTAACGCAGATGCAGCGAACGCTCGGCGTATTCTCAACAAGATTAACCGACAGATTCAATCTCGTGGATGGACGTTCAACATTGAGGAAGGCATAACGCTACTACCTGATGTTTACTCCAACCTGATTGTATACAGTGACGGCTATTTATCCCTAATGTCTACTTCCGGTCAATCCATCTACGTTAACCGAGGTGGCTAtgtg                                                                                                                                                                                                                                              <  1:268840/298‑1 (MQ=255)`  
                                                          `acaggATATGAACGTTGAGACTGCCGCTGAGTTATCAGCTGTGAACGACATTCTGGCGTCTATCGGTGAACCTCCGGTATCAACGCTGGAAGGTGACGCTAACGCAGATGCAGCGAACGCTCGGCGTATTCTCAACAAGATTAACCGACAGATGCAAGCTCGTGGATGGACGTTCAACATTGAGGAAGGCATAACGCTACTACCTGATGTTGACTCCAACCTGATTGTATACAGTGACGGCTATTTATCCCTAATGTCTACTTCCGGTCAATCCATCTACGTTAACCGAGGTGGCTAtgtg                                                                                                                                                                                                                                              <  1:181736/297‑1 (MQ=255)`  
                                                            `gcGATATGAACGTTGAGACTGCCGCTGAGTTATCAGCTGTGAACGACATTCTGGCGTCTATCGGTGAACCTCCGGTATCAACGCTGGAAGGTGACGCTAACGCAGATGCAGCGAACGCTCGGCGTATTCTCAACAAGATTAACCGACAGATTCAATCTCGTGGATGGACGTTCAACATTGAGGAAGGAATAACGCTACTACCTGATGTTTACTCCAACCTGATTGTATACAGTGACGGCTATTTATCCCTAATGTCTACTTCCGGTCAATCCATCTACGTTAACCGAGGTGGCTATGTGTa                                                                                                                                                                                                                                            <  1:102744/300‑1 (MQ=255)`  
                                                               `atatGAACGTTGAGACTGCCGCTGAGTTATCAGCTGTGAACGACATTCTGGCGTCTATCGGTGAACCTCCGGTATCAACGCTGGAAGGTGACGCTAACGCAGATGCAGCGAACGCTCGGCGTATTCTCAACAAGATTAACCGACAGATTCAATCTCGTGGATGGACGTTCAACATTGGGGAAGGCATAACGCTACTACCTGATGTTTACTCCAACCTGATTGTATACAGTGACGGCTATTTATCCCTAATGTCTACTTCCGGTCAATCCATCTACGTTAACCGAGGTGGCTATGTGTATGa                                                                                                                                                                                                                                         >  1:86575/1‑301 (MQ=255)`  
                                                               `atatGAACGTTGAGACTGCCGCTGAGTTATCAGCTGTGAACGACATTCTGGCGTCTATCGGTGAACCTCCGGTATCAACGCTGGAAGGTGACGCTAACGCAGATGCAGCGAACGCTCGGCGTATTCTCAACAAGATTAACCGACAGATTCAATCTCGTGGATGGACGTTCAACATTGAGGAAGGCATAACGCTACTACCTGATGTTTACTCCACCCTGATTGTATACAGTGACGGCTATTTATCCCTAATGTCTACTTCCGGTCACTCCATCTACGTTAACCGAGGTGCCTCTGTGTAtta                                                                                                                                                                                                                                         >  1:325749/1‑299 (MQ=255)`  
                                                                `tatGAACGTTGGGACTGCCGCTGAGTTATCAGCTGTGAACGACATTCTGGCGTCTATCGGTGAACCTCCGGTATCAACGCTGGAAGGTGACGCTAACGCAGATGCAGCGAACGCTCGGCGTATTCTCAACAAGATTAACCGACAGATTCAATCTCGTGGATGGACGTTCAACATTGAGGAAGGCATAACGCTACTACCTGATGTTTACTCCAACCTGATTGTATACAGTGACGGCTATTTATCCCTAATGTCTACTTCCGGTCAATCCATCTACGTTAACCGAGGTGGCTATGTGTATGAc                                                                                                                                                                                                                                        <  1:223045/301‑1 (MQ=255)`  
                                                                `taataaCGTTGAGACTGCCGCTGAGTTATCAGCTGTGAACGACATACTGGCGTCTATCGGTGAACCTCCGGTATCAACGCTGGAAGGTGACGCTAACGCAGATGCAGCGAACGCTCGGCGTATTCTCAACAAGATTAACCGACAGATTCCATCTCGTGGATGGACGTTCAACATTGAGGAAGACATAACGCTACTACCTGATGTTTACTCCAACCTGATTGTATACAGTGACGGCTATTTATCCCTAATGTCTACTTCCGGTCAATCCATCTACGTTACCCGAGGTGGCTATGTGTATGAc                                                                                                                                                                                                                                        <  1:142589/297‑1 (MQ=255)`  
                                                                `aatGAACGTTGAGACTGCCGCTGAGTTATCATCTGTGAACGACATTCGGGCGCCTATGCGCGAACCTCCGGTATCAACGCTGGAAGGTGACGCTAACGCAGATGCAGCGAACGCTCGGCGTATTCTCAACAAGATTAACCGACAGATTCAATCTCGTGGATGGACGTTAAACATTGAGGAAGGCATAACGCTACTACCTGATGTTTACTCCAACCTGATTGTATACAGTGACGGCTATTTATCCCTAATGTCTACTTCCGGTCAATCCATCTACGTTAACCGAGGTGGCTATGTGTATGAc                                                                                                                                                                                                                                        <  1:32960/300‑1 (MQ=255)`  
                                                                `aatGAACGTGGAGACGTCCACTGAGTTATCAGCTGTGAACGACATCCTGGCGTCTATCGGTGAAACTCCGGAATCAAAGATGGAAGGTGACGCTAACGCAGATGCAGCGAACGCTCGGCGTATTCTCAACAAGATTAACTGACATATTCAATCTCGTGGATGGTCGTTCAACATAGAGGAAGGCATAACGCTACTACCTTATGTTTACTCCAACCTGATTGTATACAGTGACGGCTATTTATCCCTAATGTCTACTTCCGGTCAATCCATCTACGTTAACCGAGGTGGCTATGTGTATGAc                                                                                                                                                                                                                                        <  1:161107/300‑1 (MQ=255)`  
                                                                   `agatgtgtataagagacaggAGTTATCAGCTGTGAACGACATGCTGGCGTCTATCGGTGAACCTCCGGTATCAACGCTGGAAGGTGACGCTAACGCAGATGCAGCGAACGCTCGGCGTATTCTCAACAAGATTAACCGACAGATTCAATCTCGTGGATGGACGTTCAACATTGAGGAAGGCATAACGCTACTACCTGATGTTTACTCCAACCTGATTGTATACAGTGACGGCTATTTATCCATAATGTCTACTTCCGGTCAATCCATCTACGTTAACCGAGGTGGCTATGTGTATGAcctg                                                                                                                                                                                                                                     <  1:42767/282‑3 (MQ=255)`  
                                                                        `tgtgtataagagacaggTTATCAGCTGGGAACGACATTCTGGCGTCTATCGGTGAACCTCCGGTATCAACGCTGGAAGGTGACGCTAACGCAGATGCAGCGAACGCTCGGCGTATTCTCAACAAGATTAACCGACAGATTCAATCTCGTGGATGGACGTTCAACATTGAGGAAGGCATAACGCTACTACCTGATGTTTACTCCAACCTGATTGTATACAGTGACGGCTATTTATCCCTAATGTCTACTTCCGGTCAATCCATCTACGTTAACCGAGGTGGCTATGTGTATGACCAAACGAg                                                                                                                                                                                                                                <  1:73781/285‑1 (MQ=255)`  
                                                                              `gacaggCTGAGTTATCAGCTGTGAACGACATTCTGGCGTCTATCGGTGAACCTCCGGTATCAACGCTGGAAGGTGACGCTAACGCAGATGCAGCGAACGCTCGGCGTATTCTCAACAAGATTAACCGACAGATTCAATCTCGTGGATGGACGTTCAACATTGAGGAAGGCATAACGCTACTACCTGATGTTTACTCCAACCTGATTGTATACAGTGACGGCTATTTATCCCTAATGTCTACTTCCGGTCAATCCATCTACGTTAACCGAGGTGGCTATGTGTATGACCGAACGAGTCAATc                                                                                                                                                                                                                          <  1:184684/296‑1 (MQ=255)`  
                                                                              `cTGCCGACGAGTTACCAGCTGTGAACGACATTCTGGCGTCTATCGGTGAACCTCCGGTATCAACGATGGAAGGTGACGCTAACGCAGATGCAGCGAACGCTCGGCGTATTCTCAACAAGATTAACCGACAGATTCAATCTCGTGGATGGAAGTTCAACATTGAGGAAGGCATAACGCTACTACCTGATGTTTACTCCAACCTGATTGTATACAGTGACGGCTATTTATCCCTAATGTCTACTTCCGGTCAATCCATCTACGTTAACCGAGGTGGCTATGTGTATGACCGAACGAGTCAATc                                                                                                                                                                                                                          <  1:125074/301‑1 (MQ=255)`  
                                                                               `tGCCGCTGAGTTATCAGCTGTGAACGACATTCTGGCGTCTATCGGTGAACCTCCGGTATCAACGCTGGAAGGTGACGCTAACGCAGATGCAGCGAACGCTCGGCGTATTCTCAACAAGATTAACCGACAGATTCAATCTCGTGGATGGACGTTCAACATTGAGGAAGGCATAACGCTACTACCTGATGTGTACTCCAACATGATTGTATACAGTGACGGCTATTTATCCCTAATGTCTACTTCCGGTCAATCCATCTACGTTAACCGAGGTGGCTATGTGTATGACCGAACGAGTCAATCa                                                                                                                                                                                                                         <  1:23116/301‑1 (MQ=255)`  
                                                                                      `gAGTTATCAGCTGTGAACGACATTCTGGCGTCTATCGGTGAACCTCCGGTATCAACGCTGGAAGGTGACGCTAACGCAGATGCAGCGAACGCTCGGCGTATTCTCAACAAGATTAACCGACAGATTCAATCTCGTGGATGGACGTTCAACATTGAGGAAGGCATAACGCTACTACCTGATGTTTACTCCAACCTGATTGTATACAGTGACGGCTATTTATCCCTAATGTCTACTTCCGGTCAATCCATCTACGTTAACCGAGGTGGCTATGTGTATGACCGAACGAGTCAATCAGACCGCt                                                                                                                                                                                                                  >  1:218392/1‑301 (MQ=255)`  
                                                                                        `gTTATCAGCTGTGAACGACATTCTGGCGTCTATCGGTGAACCTCCGGTATCAACGCTGGAAGGTGACGCTAACGCAGATGCAGCGAACGCTCGGCGTATTCTCAACAAGATTAACCGACAGATTCAATCTCGTGGATGGACGTTCAACATTGAGGAAGGCATAACGCTACTACCTGATGTTTACTCCAACCTGCTTGTATACAGTGACGGCTATTTATCCCTAATGTCTACTTCCGGTCAATCCATCTACGTTAACCGAGGTGGCTATGTGTATGACCGAACGAGTCAATCAGACAGCttt                                                                                                                                                                                                                >  1:247996/1‑301 (MQ=255)`  
                                                                                                  `gtgAACGACATTCTGGCGTCTATCGGTGAACCTCCGGTATCAACGCTGGAAGGTGACGCTAACGCCGATGCAGCGAACGCTCGGCGTATTCTCAACAAGATTAACCGACAGATTCAATCTCGTGGATGGACGTTCAACATTGAGGAAGGCATAACGCTACTACCTGATGTTTACTCCAACCTGATTGTATACAGTGACGGCTATTTATCCCTAATGTCTACTTCCGGTCAATCCATCTACGTTAACCGAGGTGGCTATGTGTATGACCGAACTAGTCAATCAGACCGCTTTTActcctgtc                                                                                                                                                                                                      >  1:336035/1‑296 (MQ=255)`  
                                                                                                        `gACATTCTGGCGTCTATCGGTGAACCTCCGGTATCAACGCTGGAAGGTGACGCTAACGCAGATGCAGCGAACGCTCGGCGTATTCTCAACAAGATTAACCGACAGATTCAATCTCGTGGATGGACGTTCAACATTGAGGAAGGCATAACGCTACTACCTGATGTTTACTCCAACCTGATTGTATACAGTGACGGCTATTTATCCCTAATGTCTACTTCCGGTCAATCCATCTACGTTAACCGAGGTGGCTATGTGTATGACCGAACGAGTCAATCAGACCGCTTTGACTCTGGTATTACtg                                                                                                                                                                                                <  1:256960/301‑1 (MQ=255)`  
                                                                                                        `gACATTCTGGCGTCTATCGGTGAACCTCCGGTATCAACGCTGGAAGGTGACGCTAACGCAGATGCAGCGAACGCTCGGCGTATTCTCAACAAGATTAACCGACAGATTCAATCTCGTGGATGGACGTTCAACATTGAGGAAGGCATAACGCTACTACCTGATGTTTACTCCAACCTGATTGTATACAGTGACGGCTATTTATCCCTAATGTCTACTTCCGGTCAATCCATCTACGTTAACCGAGGTGGCTATGTGTATGACCGAACGAGTCAATCAGACCGCTTTGACTCTGGTATTACtg                                                                                                                                                                                                <  1:16763/301‑1 (MQ=255)`  
                                                                                                             `tCTGGCGTCTATCGGTGAACCTCCGGTATCAACGCTGGAAGGTGACGCTAACGCAGATGCAGCGAACGCTCGGCGTATTCCCAACAAGAGTAACCGACAGATTCAATCTCGTGGATGGACGTTCAACATTGAGGAAGGCATAACGCTACTACCTGATGTTTACTCCAACCTGATTGTATACAGTGACGGCTATTTATCCCTAATGTCTACTTCCGGTCAATCCATCTACGTTAACCGAGGTGGCTATGTGTATGACCGAACGAGTCAATCAGACCGCTTTGACTCTGGTATTACTGTGAAc                                                                                                                                                                                           <  1:245248/301‑1 (MQ=255)`  
                                                                                                                   `gTCTATCGGTGAACCTCCGGTATCAACGCTGGAAGGTGACGCTAACGCAGATGCAGCGAACGCTCGGCGTATTCTCAACAAGATTAACCGACAGATTCAATCTCGTGGATGGACGTTCAACATTGAGGAAGGCATAACGCTACTACCTGATGTTTACTCCAACCTGATTGTATACAGTGACGGCTATTTATCCCTAATGTCTACTTCCGGTCAATCCATCTACGTTAACCGAGGTGGCTATGTGTATGACCGAACGAGTCAATCAGACCGCTTTGACTCTGGTATTACTTTGTACattatt                                                                                                                                                                                     >  1:118006/1‑301 (MQ=255)`  
                                                                                                                     `cacgctgttacccTCCGGTATCAACGCTGGAAGGTGACGCTAACGCAGATGCAGCGAACGCTCGGCGTATTCTCAACAAGATTAACCGACAGATTCAATCTCGTGGATGGACGTTCAACATTGAGGAAGGCATAACGCTACTACCTGATGTTTACTCCAACCTGATTGTATACAGTGACGGCTATTTATCCCTAATGTCTACTTCCGGTCAATCCATCTACGTTAACCGAGGTGGCTATGTGTATGACCGAACGAGTCAATCAGACCGCTTTGACTCTGGTATTACTGTGAACATTATTCg                                                                                                                                                                                   <  1:17861/290‑1 (MQ=255)`  
                                                                                                                               `aCCTCTGGTATCAACGCTGGAAGGTGACGCTAACGCAGATGCAGCGAACGCTCGGCGTATTCTCAACAAGATTAACCGACAGATTCAATCTCGTGGATGGACGTTCAACATTGAGGAAGGCATAACGCTACTACCTGATGTTTACTCCAACCTGATTGTATACAGTGACGGCTATTTATCCCTAATGTCTACTTCCGGTCAATCCATCTACGTTAACCGAGGTGGCTATGTGTATGACCGAACGAGTCAATCAGACCGCTTTGCCTCTGGTATTACTGTGAACATTATTCGTCTCCGCGAc                                                                                                                                                                         >  1:51691/1‑301 (MQ=255)`  
                                                                                                                                  `tCCGGTATCAACGCTGGAAGGTGACGCTAACGCAGATGCAGCGAACGCTCGGCGTATTCTCAACAAGATTAACCGACAGATTCAATCTCGTGGATGGACGTTCAACATTGAGGAAGGCATAACGCTACTACCTGATGTTTACTCCAACCTGATTGTATACAGTGACGGCTATTTATCCCTAATGTCTACTTCCGGTCAATCCATCTACGTTAACCGAGGTGGCTATGTGTATGACCGAACGAGTCAATCAGACCGCTTTGACTCTGGTATTACTGTGAACATTATTCGTCTCCGCGACTAc                                                                                                                                                                      >  1:266033/1‑301 (MQ=255)`  
                                                                                                                                         `taagagacagAAGGTGACGCTAACGCAGATGCAGCGAACGCTCGGCGTATTCTCAACAAGATTAACCGACAGATTCAATCTCGTGGATGGACGTTCAACATTGAGGAAGGCATAACGCTACTACCTGATGTTTACTCCAACCTGATTGTATACAGTGACGGCTATTTATCCCTAATGTCTACTTCCGGTCAATCCATCTACGTTAACCGAGGTGGCTATGTGTATGACCGAACGAGTCAATCAGACCGCTTTGACTCTGGTATTACTGTGAACATTATTCGTCTCCGCGACTACGATgaga                                                                                                                                                               <  1:54292/292‑1 (MQ=255)`  
                                                                                                                                            `aCGCTGGAAGGTGACGCTAACGCAGATGCAGCGAACGCTCGGCGTATTCTCAACAAGATTAACCGACAGATTCAATCTCGTGGATGGACGTTCAACATTGAGGAAGGCATAACGCTACTACCTGATGTTTACTCCAACCTGATTGTATACAGTGACGGCTATTTATCCCTAATGTCTACTTCCGGTCAATCCATCTACGTTAACCGAGGTGGCTATGTGTATGACCGAACGAGTCAATCAGACCGCTTTGACTCTGGTATTACTGTGAACATTATTCGTCTCCGCGACTActgtctcttac                                                                                                                                                            >  1:48637/1‑291 (MQ=255)`  
                                                                                                                                            `aCGCTGGAAGGTGACGCTAACGCAGATGCAGCGAACGCTCGGCGTATTCTCAACAAGATTAACCGACAGATTCAATCTCGTGGATGGACGTTCAACATTGAGGAAGGCATAACGCTACTACCTGATGTTTACTCCAACCTGATTGTATACAGTGACGGCTATTTATCCCTAATGTCTACTTCCGGTCAATCCATCTACGTTAACCGAGGTGGCTATGTGTATGACCGAACGAGTCAATCAGACCGCTTTGACTCTGGTATTACTGTGAACATTATTCGTCTCCGCGACTACGATGAGATGc                                                                                                                                                            >  1:280117/1‑301 (MQ=255)`  
                                                                                                                                               `cTGGAAGGTGACGCTAACGCAGATGCAGCGAACGCTCGGCGTATTCTCAACAAGATTAACCGACAGATTCAATCTCGTGGATGGACGTTCAACATTGAGGAAGGCATAACGCTACTACCTGATGTTTACTCCAACCTGATTGTATACAGTGACGGCTATTTATCCCTAATGTCTACTTCCGGTCAATCCATCTACGTTAACCGAGGTGGCTATGTGTATGACCGAACGAGTCAATCAGACCGCTTTGACTCTGGTATTACTGTGAACATTATTCGTCTCCGCGACTACGATGAGATGCCTg                                                                                                                                                         <  1:323814/301‑1 (MQ=255)`  
                                                                                                                                                      `gTGACGCTAACGCAGATGCAGCGAACGCTCGGCGTATTCTCAACAAGATTAACCGACAGATTCAATCTCGTGGATGGACGTTCAACATTGAGGAAGGCATAACGCTACTACCTGATGTTTACTCCAACCTGATTGTATACAGTGACGGCTATTTATCCCTAATGTCTACTTCCGGTCAATCCATCTACGTTAACCGAGGTGGCTATGTGTATGACAGAACGAGTCAATCAGACCGCTTTGACTCTGGTATTACTGTGAACATTATTCGTCTCCGCGACTACGATGAGATGCCTGAGTGCtt                                                                                                                                                  >  1:34915/1‑301 (MQ=255)`  
                                                                                                                                                         `aCGCTAACGCAGATGCAGCGAACGCTCGGCGTATTCTCAACAAGATTAACCGACAGATTCAATCTCGTGGATGGACGTTCAACATTGAGGAAGGCATAACGCTACTACCTGATGTTTACTCCAACCTGATTGTATACAGTGACGGCTATTTATCCCTAATGTCTACTTCCGGTCAATCCATCTACGTTAACCGAGGTGGCTATGTGTATGACCGAACGAGTCAATCAGACCGCTTTGACTCTGGTATTACTGTGAACATTATTCGTCTCCGCGACTACGATGAGATGCCTGAGTGCTTCCg                                                                                                                                               <  1:284287/301‑1 (MQ=255)`  
                                                                                                                                                                         `ggctcggagatgtgtataagagacagaGATTAACCGACAGATTCAATCTCGTGGATGGACGTTCAACATTGAGGAAGGCATAACGCTACTACCTGATGTTTACTCCAACCTGATTGTATACAGTGACGGCTATTTATCCCTAATGTCTACTTCCGGTCAATCCAACTACGTTAACCGAGGTGGCTATGTGTATGACCGAACGAGTCAATCAGACCGCTTTGACTCTGGTATTACTGTGAACATTATTCGTCTCCGCGACTACGATGAGATGCCTGAGTGCTTCCGTTACTGGATTGTCAcc                                                                                                                               <  1:154461/275‑1 (MQ=255)`  
                                                                                                                                                                                   `cGGCGTATTCTCAACAAGATTAACCGACAGATTCAATCTCGTGGATGGACGTTCAACATTGAGGAAGGCATAACGCTACTACCTGATGTTTACTCCAACCTGATTGTATACAGTGACGGCTATTTATCCCTAATGTCTACTTCCGGTCAATCCATCTACGTTAACCGAGGTGGCTATGTGTATGACCGAACGAGTCAATCAGACCGCTTTGACTCTGGTATTACTGTGAACATTATTCGTCTCCGCGACTACGATGAGATGCCTGAGTGCTTCCGTTACTGGATTgctgtctctcatacac                                                                                                                     >  1:339189/1‑286 (MQ=255)`  
                                                                                                                                                                                        `tataagagacagGATTAACCGACAGATTCAATCTCGTGGATGGACGTTCAACATTGAGGAAGGCATAACGCTACTACCTGATGTTTACTCCAACCTGATTGTATACAGTGACGGCTATTTATCCCTAATGTCTACTTCCGGTCAATCCATCTACGTTAACCGAGGTGGCTATGTGTATGACCGAACGAGTCAATCATACCGCTTTGACTCTGGTATTACTGTGAACATTATTCGTCTCCGCGACTACGATGAGATGCCTGAGTGCTTCCGTTACTGGATTGTCACCAAGGCTTCCCGTCAg                                                                                                                <  1:149481/293‑1 (MQ=255)`  
                                                                                                                                                                                         `aTTCTCAACAAGATTAACCGACAGATTCAATCTCGTGGATGGACGTTCAACATTGAGGAAGGCATAACGCTACTACCTGATGTTTACTCCAACCTGATTGTATACAGTGACGGCTATTTATCCCTAATGTCTACTTCCGGTCAATCCATCTACGTTAACAGAGGTGGCTATGTGTATGACCGAACGAGTCAATCAGACCGCTTTGACTCTGGTATTACTGTGAACATTATTCGTCTACGCGACTACGATGAGATGCCTGAGTGCTTCAGTTACTGGATTGTAACCAAGGcctgtctcttat                                                                                                               >  1:263245/1‑290 (MQ=255)`  
                                                                                                                                                                                                                 `aTTCAATCTCGTGGATGGACGTTCAACATTGAGGAAGGCATAACGCTACTACCTGATGTTTACTCCAACCTGATTGTATACAGTGACGGCTATTTATCCCTAATGTCTACTTCCGGTCAATCCATCTACGTTAACCGAGGTGGCTATGTGTATGACCGAACGAGTCAATCAGACCGCTTTGACTCTGGTATTACTGTGAACATTATTCGTCTCCGCGACTACGATGAGATGCCTGAGTGCTTCCGTTACTGGATTGTCACCAAGGCTTCCCGTCAGTTCAACAACCGATTCTTTGGGGCAc                                                                                       >  1:33309/1‑301 (MQ=255)`  
                                                                                                                                                                                                                 `aTTCAATCTCGTGGATGGACGTTCAACATTGAGGAAGGCATAACGCTACTACCTGATGTTTACTCCAACCTGATTGTATACAGTGACGGCTATTTATCCCTAATGTCTACTTCCGGTCAATCCATCTACGTTAACCGAGGTGGCTATGTGTATGACCGAACGAGTCAATCAGACCGCTTTGACTCTGGTATTACTGTGAACATTATTCGTCTCCGCGACTACGATGAGATGCCTGAGTGCTTCCGTTACTGGATTGTCACCAAGGCTTCCCGTCAGTTCAACAACCGATTCTTTGGGGCAc                                                                                       >  1:209054/1‑301 (MQ=255)`  
                                                                                                                                                                                                                 `aTTCAATCTCGTGGATGGACGTTCAACATTGAGGAAGGCATAACGCTACTACCTGATGTTTACTCCAACCTGATTGTATACAGTGACGGCTATTTATCACTAATGTCTACTTCCGGTCAATCCATCTACGTTAACCGAGGTGGCTATGTGTATGACCGAACGAGTCAATCAGACCGCTTTGACTCTGGTATTACTGTGAACATTATTCGTCTCCGCGACTACGATGAGATGCCTGAGTGCTTCCGTTACTGGATTGTCACCAAGGCTTCCCGTCAGTTCAACAACCGATTCTTTGGGGCAc                                                                                       <  1:277197/301‑1 (MQ=255)`  
                                                                                                                                                                                                                      `aTCTCGTGGATGGACGTTCAACATTGAGGAAGGCATAACGCTACTACCTGATGTTTACTCCAACCTGATTGTATACAGTGACGGCTATTTATCCCTAATGTCTACTTCCGGTCAATCCATCTACGTTAACCGAGGTGGCTATGTGTATGACCGAACGAGTCAATCAGACCGCTTTGACTCTGGTATTACTGTGAACATTATTCGTCTCCGCGACTACGATGAGATGCCTGAGTGCTTCCGTTACTGGATTGTCACCAAGGCTTCCCGTCAGTTCAACAACCGATTCTTTGGGGCACCGGaa                                                                                  >  1:200773/1‑301 (MQ=255)`  
                                                                                                                                                                                                                        `ctcGTGGATGGACGTTCAACATTGAGGAAGGCATAACGCTACTACCTGATGTTTACTCCAACCTGATTGTATACAGTGACGGCTATTTATCCCTAATGTCTACTTCCGGTCAATCCATCTACGTTAACCGAGGTGGCTATGTGTATGACCGAACGAGTCAATCAGACCGCTTTGACTCTGGTATTACTGTGAACATTATTCGTCTCCGCGACTACGATGAGATGGCTGAGTGCTTCCGTTACTGGATTGTCACCAAGGCTTCCCGTCAGTTCAACAACCGATGCTTGGGGGCACCGGAAgg                                                                                >  1:198349/1‑300 (MQ=255)`  
                                                                                                                                                                                                                        `ctcGTGGATGGACGTTCAACATTGAGGAAGGCATAACGCTACTACCTGATGTTTACTCCAACCTGATTGTATACAGTGACGGCTATTTATCCCTAATGTCTACTTCCGGTCAATCCATCTACGTTAACCGAGGTGGCTATGTGTATGACCGAACGAGTCAATCAGACCGCTTTGACTCTGGTATTACTGTGAACATTATTCGTCTCCGCGACTACGATGAGATGCCTTAGTGCTTCCGTTACTGGATTGTCACCAAGGCTTCCCGTCAGTTCAAAAACCGATTATTTGGGGCACCGGAAgg                                                                                >  1:18813/1‑300 (MQ=255)`  
                                                                                                                                                                                                                          `cGTGGATGGACGTTCAACATTGAGGATGGCATAACGCTACTACCTGATGTTTACTCCAACCTGATTGTATACAGTGACGGCTTTTTATCCCTAATGTCTACTTCCGGTCAATCCATCTCCGTTAACCTAGGTTGCTCTTTGTATGTCCGAACGAGTCAATCAGACCGCTTTGACTCTGGTATTACTGTGTACATTATTCGTCTCCGCGACTACTATGAGATGCCTGCTTGCTTCCGTTACTGTATTGTCACCTAGGCTTCCCGTCAGTTCAACAACCTCTTCTTTTGGGCACCGGCAGTag                                                                              >  1:292107/1‑301 (MQ=255)`  
                                                                                                                                                                                                                          `cGTGGATGGACGTTCAACATTGAGGAAGGCATAACGCTACTACCTGATGTTTACTCCAACCTGATTGTATACAGTGACGGCTATTTATCCCTAATGTCTACTTCCGGTCAATCCATCTACGTTAACCGAGGTGGCTATGTGTATGACCGAACGAGTCAATCAGACCGCTTTGACTCTGGTATTACTGTGAACATTATTCGTCTCCGCGACTACGATGAGATGCCTGAGTGCTTCCGTTACTGGATTGTCACCAAGGCTTCCCGTCAGTTCAACAACCGATTCTTTGGGGCACCGGAAGTag                                                                              >  1:206409/1‑301 (MQ=255)`  
                                                                                                                                                                                                                            `tggatggaCGTTCAACATTGAGGAAGGCATAACGCTACTACCTGATGTTTACTCCAACCTGATTGTATACAGTGACGGCTATTTATCCCTAATGTCTACTTCCGGTCAATCCATCTACGTTAACCGAGGTGGCTATGTGTATGACCGAACTAGTCAATCAGACCGCTTTGACTCTTGTATTACTGTGAATATTATTCGTCTCCGCGACTACGATGAGATGCCTGAGTGCTTCCGTTACTGGATTGTCACCAAGGCTTCCCGTCAGTTCAACAACCGATTCTTTGTGGCACCGGAAGTagag                                                                            <  1:294919/301‑1 (MQ=255)`  
                                                                                                                                                                                                                                   `aCGTTCAACATTGAGGAAGGCATAACGCTACTACCTGATGTTTACTCCAACCTGATTGTATACAGTGACGGCTATTTATCCCTAATGTCTACTTCCGGTCAATCCATCTACGTTAACCGAGGTGGCTATGTGTATGACCGAACGAGTCAATCAGACCGCTTTGACTCTGGTATTACTGTGAACATTATTCGTCTCCGCGACTACGATGAGATGCCTGAGTGCTTCCGTTACTGGATTGTCACCAAGGCTTCCCGTCAGTTCAACAACCGATTCTTTGGGGCACCGGAAGTAGAGGGTGTAc                                                                     >  1:288540/1‑301 (MQ=255)`  
                                                                                                                                                                                                                                     `ggtCAACATTGAGGAAGGCATAACGCTACTACCTGATGTTTACTCCAACCTGATTGTATACAGTGACGGCTATTTATCCCTAATGTCTACTTCCGGTCAATCCATCTACGTTAACCGAGGTGGCTATGTGTATGACCGAACGAGTCAATCAGACCGCTTTGACTCTGGTATTACTGTGAACATTATTCGTCTCCGCGACTACGATGAGATGCCTGAGTGCTTCCGTTACTGGATTGTCACCAAGGCTTCCCGTCAGTTCAACAACCGATTCTTTGGGGCACCGGAAGTAGAGGGTGTACTc                                                                   <  1:273490/299‑1 (MQ=255)`  
                                                                                                                                                                                                                                     `gTTCAACATTGAGGAAGGCATAACGCTACTACCTGATGTTTACTCCAACCTGATTGTATACAGTGACGGCTATTTATCCCTAATGTCTACTTCCGGTCAATCCATCTACGTTAACCGAGGTGGCTATGTGTATGACCGAACGAGTCAATCAGACCGCTTTGACTCTGGTATTACTGTGAACATTATTCGTCTCCGCGACTACGATGAGATGCCTGAGTGCTTCCGTTACTGGATTGTCACCAAGGCTTCCCGTCAGTTCAACAACCGATTCTTTGGGGCACCGGAAGTAGAGGGTGTACTc                                                                   >  1:192324/1‑301 (MQ=255)`  
                                                                                                                                                                                                                                     `gTTCAACATTGAGGAAGGCATAACGCTACTACCTGATGTTTACTCCAACCTGATTGTATACAGTGACGGCTATTTATCCCTAATGTCTACTTCCGGTCAATCCATCTACGTTAACCGAGGTGGCTATGTGTATGACCGAACGAGTCAATCAGACAGCTTTGACTCTGGTATTACTGTGAACATTATTCGTCTCCGCGACTACGATGAGATGCCTGAGTGCTTCCGTTACTGGATTGTCACCAAGGCTTCCCGTCAGTTCAACAACCGATTCTTTGGGGCACCGGAAGTAGAGGGTGTACTc                                                                   >  1:97593/1‑301 (MQ=255)`  
                                                                                                                                                                                                                                      `ctcggagatgtgtataagagacagCTACTACCTGATGTTTACTCCAACCTGATTGTATACAGTGACGGCTATTTATCCCTAATGTCTACTTCCGGTCAATCCATCTACGTTAACCGAGGTGGCTATGTGTATGACCGAACGAGTCAATCAGACCGCTTTGACTCTGGTATTACTGTGAACATTATTCGTCTCCGCGACTACGATGAGATGCCTGAGTGCTTCCGTTACTGGATTGTCACCAAGGCTTCCCGTCAGTTCAACAACCGATTCTTTGGGGCACCGGAAGTAGAGGGTGTACTcc                                                                  <  1:89687/278‑1 (MQ=255)`  
                                                                                                                                                                                                                                           `agatgtgtataagagacagCTACTACCTGATGTGTACTCCAACCTGATTGTATACAGTGACGGCTATTTATCCCTAATGGCTACTTCCGGTCAATCCATCTACGTTAACCGAGGTGGCTATGTGTATGACCGAACGAGTCAATCAGACCGCTTTGACTCTGGTATTACTGTGAACATTATTCGTCTCCGCGACTACGATGAGATGCCTGAGTGCTTCCGTTACTGGATTGTCACCAAGGCTTCCCGTCAGTTCAACAACCGATTCTTTGGGGCACCGGAAGTAGAGGGTGTACTCCaagaa                                                             <  1:67819/283‑1 (MQ=255)`  
                                                                                                                                                                                                                                                     `tgtataagatacagacCTGATGTTTACTCCAACCTGATTGTATACAGTGACGGCTATTTATCCCTAATGTCTACTTCCGGTCAATCCATCTACGTTAACCGAGGTGGCTATGTGTATGACCGAACGAGTCAATCAGACCGCTTTGACTCTGGTATTACTGTGAACATTATTCGTCTCCGCGACTACGATGAGATGCCTGAGTGCTTCCGTTACTGGATTGTCACCAAGGCTTCCCGTCAGTTCAACAACCGATTCTTTGGGGCACCGGAAGTAGAGGGTGTACTCCAAGAAGAGGAAGATg                                                   <  1:95035/287‑1 (MQ=255)`  
                                                                                                                                                                                                                                                            `cGCTACTACCTGATGTTTACTCCAACCTGATTGTATACAGTGACGGCTATTTATCCCTAATGTCTACTTCCGGTCAATCCATCTACGTTAACCGAGGTGGCTATGTGTATGACCGAACTAGTCAATCAGACCGCTTTGACTCTGGTATTACTGTGAACATTATTCGTCTCCGCGACTACGATGAGATGCCTGAGTGCTTCCGTTACTGGATTGTCACCAAGGCTTCCCGTCAGTTCAACAACCGATTCTTTGGGGCACCGGACGTAGAGGGTGTACTCCAAGAAGAGGAAGATGAAGCTAg                                            >  1:242149/1‑301 (MQ=255)`  
                                                                                                                                                                                                                                                              `ctactaCCTGATGTTTACTCCAACCTGTTTGTATACAGTGACGGCTATTTATCCCTAATGTCTACTTCCGGTCAATCCATCTACGTTAACCGAGGTGGCTATGTGTATGACCGAACGAGTCAACCAGACCGCTTTGACTCTGGTATTACTGTGAACATTATTCGTCTCCGCGACTACGATGAGATGCCTGAGTGCTTCCGTTACTGGATTGTCACCAAGGCTTCCCGTCAGTTCAACAACCGATTCTTTGGTGCACCGGAAGGATAGGGTGTACTCCAAGAAGAGGAAGATGAGGCTAGAc                                          >  1:220825/1‑301 (MQ=255)`  
                                                                                                                                                                                                                                                               `tactacCTGATGTTTACTCCAACCTGATTGTATACAGTGACGGCTATTTATCCCTAATGTCTACTTCCGGTCAATCCATCTACGTTAACCGAGGTGGCTATGTGTATGACCGAACGAGTCAATCAGACCGCTTTGACTCTGGTATTACTGTGAACATTATTCGTCTCCGCGACTACGATGAGATGCCTGAGTGCTTCCGTTACTGGATTGTCACAAAGGCTTCCCGTCAGTTCAACAACCGATTCTTTGGGGCACCGGAAGTAGAGGGTGTACTCCAAGAAGAGGAAGATGAAGCTAGACg                                         <  1:151873/301‑1 (MQ=255)`  
                                                                                                                                                                                                                                                               `ggagatgtgtataagagacagaCCTGATTGTATACAGTGACGGCTATTTATCCCTAATGTCTACTTCCGGTCAATCCATCTACGTTAACCGAGGTGGCTATGTGTATGACCGAACGAGTCAATCAGACCGCTTTGACTCTGGTATTACTGTGAACATTATTCGTCTCCGCGACTACGATGAGATGCCTGAGTGCTTCCGTTACTGGATTGTCACCAAGGCTTCCCGTCAGTTCAACAACCGATTCTTTGGGGCACCGGAAGTAGAGGGTGTACTCCAAGAAGAGGAAGATGAGGCTAGACg                                         <  1:105568/280‑1 (MQ=255)`  
                                                                                                                                                                                                                                                                `actacCTGATGTTTACTCCAACCTGATTGTATACAGTGACGGCTATTTATCCCTAATGTCTACTTCCGGTCAATCCATCTACGTTAACCGAGGTGGCTATGTGTATGACCGACCGAGTCAATCAGACCGCTTTGACTCTGGTATTACTGTGAACATTATTCGTCTCCTCGACTACGATGAGATGCCTGAGTGCTTCCGTTACTGGATTGTCACCCAGGCTCCCCGTCAGTTCAACAACCGATTCTTTGGGGCACCGGATGTATAGGGTGTCCTCAAagaagactttctcttatacacatct                                        >  1:313862/1‑282 (MQ=255)`  
                                                                                                                                                                                                                                                                  `tacCTGATGTTTACTCCAACCTGATTGTATACAGTGATGGCTATTTATCCCTAATGTCTACTTCCGGTCAATCCATCTACGTTAACCGAGGTGGCTATGTGTATGACCGAACGAGTCAATCAGACCGCTTTGACTCTGGTATTACTGTGAACATTATTCGTCTCCGCGACTACGATGAGATGCCTGAGTGCTTCCGTTACTGGATTGTCACCAAGGCTTCCCGTCAGTTCAACAACCGATTCTTTGGGGCACCGGAAGTAGAGGGTGTACTCCAAGAAGAGGAAGATGAGGCTAGACGtct                                      >  1:76251/1‑301 (MQ=255)`  
                                                                                                                                                                                                                                                                   `acCTGATGTTTACTCCAACCTGATTGTATACAGTGACGGCTATTTATCCCTAATGTCTACTTCCGGTCAATCCATCTACGTTAACCGAGGTTGCTATGTGTATGACCGAACGAGTCAATCAGACCGCTTTGACTCTGGTATTACTGTGAACATTATTCGTCTCCGCGACTACGATGAGATGCCTGAGTGCTTCCGTTACTGGATTGTCACCAAGGCTTCCCGTCAGTTCAACAACCGATTCTTTGGGGCACCGGAAGTAGAGGGTGTACTCCAAGAAGAGGAAGATTAGGCTAGACGtctc                                     >  1:238779/1‑301 (MQ=255)`  
                                                                                                                                                                                                                                                                    `ccTGATGTTTACTCCAACCTGATTGTATACAGTGACGGCTATTTATCCCTAATGTCTACTTCCGGTCAATCCATCTACGTTAACCGAGGTGGCTATGTGTATGACCGAACGAGTCAATCAGACCGCTTTGACTCTGGTATTACTGTGAACATTATTCGTCTCCGCGACTACGATGAGATGCCTGAGTGCTTCCGTTACTGGATTGTCACCAAGGCTTCCCGTCAGTTCAACAACCGATTCTTTGGGGCACCGGAAGTAGAGGGTGTACTCCAAGAAGAGGAAGATGAGGATAGACGTctct                                    >  1:302311/1‑301 (MQ=255)`  
                                                                                                                                                                                                                                                                     `cTGATGTTTACTCCAACCTGATTGTATACAGTGACGGCTATTTATCCCTAATGTCTACTTCCGGTCAATCCATCTACGTTAACCGAGGTGGCTATGTGTATGACCGAACGAGTCAATCAGACCGCTTTGACTCTGGTATTACTGTGAACATTATTCGTCTCCGCGACTACGATGAGATGCCTGAGTGCTTCCGTTACTGGATTGTCACCAAGGCTTCCCGTCAGTTCAACAACCGATTCTTTGGGGCACCGGAAGTAGAGGGTGTACT‑CAAGAAGAGGAAGATGAGGCTAGACGTCTCTGc                                  >  1:307918/1‑301 (MQ=255)`  
                                                                                                                                                                                                                                                                          `gtgtataagatacagATTGTATACAGTGACGGCTATTTATCCCTAATGTCTACTTCCGGTCAATCCATCTACGTTAACCGATGTGGCTATGTGTATGACCGAACGAGTCAATCAGACCGCTTTGACTCTGGTATTACTGTGAACATTATTCGTCTCCGCGACTACGATGAGATGCCTGAGTGCTTCCGATACTGGATTGTCACCAAGGCTTCCCGTCAGTTCAACAACCGATTCTTTTGGGCACCGGAAGTAGAGGGTGTACTCCAAGAAGAGGAAGATGAGGCTAGACGTCTCTGCATgg                              <  1:100028/287‑1 (MQ=255)`  
                                                                                                                                                                                                                                                                          `gTTTACTCCAACCTGATTGTATACAGTGACGGCTATTTATCCCTAATGTCTACTTCCGGTAAATCCATCTACGTTAACCGAGGTGGCTATGTGTATGACCGAACGAGTCAATCAGACCGCTTTGACTCTGGTATTACTGTGAACATTATTCGTCTCCGCGACTACGATGAGATGCCTGAGTGCTTCCGTTACTGGATTGTCACCAAGGCTTCCCGTCAGTTCAACAACCGATTCTTTGGGGCACCGGAAGTAGAGGGTGTACTCCAAGAAGAGGAAGATGAGGCTAGACgctgtctcttat                              >  1:60605/1‑290 (MQ=255)`  
                                                                                                                                                                                                                                                                                  `cagccTGATTGTATACAGTGACGGCTATTTATCACGAATGTCTACTTCAGGTAAATCCATCTACGTTAACAGAGGTGGCTATGTGTAGGACCGAACGAGTCAATCAGACCGCTTTGACTCTGGTATTACTGTGAACATTATTCGTCTCCGCGACTACGATGAGATGCCTGAGTGCTTCCGTTACTGGATTGTCAACAAGGCTTCCCGTCAGTTCAACAACCGATTCTTTGGGGCACCGGAAGTAGAGGGTGTACTCCAAGAAGAGGAAGATGAGGCTAGACGTCTCTGCATGGAGTATgag                      <  1:84116/298‑1 (MQ=255)`  
                                                                                                                                                                                                                                                                                  `aacagtGATTGTATACAGTGACGGCTATTTATCCCTAATGTCTACTTCCGGTCAATCCATCTACGTTAACCGAGGTGGCTATGTGTATGACCGAACGAGTCAATCAGACCGCTTTGACTCTGGTATTACTGTGAACATTATTCGTCTCCGCGACTACGATGAGATGCCTGAGTGCTTCCGTTACTGGATTGTCACCAAGGCTTCCCGTCAGTTCAACAACCGATTCTTTGGGGCACCGGAAGTAGAGGGTGTACTCCAAGAAGAGGAAGATGAGGCTAGACGTCTCTGCATGGAGTAGgag                      <  1:253493/296‑1 (MQ=255)`  
                                                                                                                                                                                                                                                                                   `gacctggtgttataCAGGGACGGCTATTGATCACTAATGGCTACTTCCGGTCAATTCATCTATGTTAACCGAGGTGGCTATGTGTATGACCGAACGAGTAAATCAGACCGTGTTGACTCTGGTATTACTGTGAACATTATTCGTCTCCGCGACTACGATGAGATGCCTGAGTGCTTCCGTTACTGGATTGTCACCAAGGCTTCCCGTCAGTTCAACAACCGATTCTTTGGGGCACAGGAAGTAGAGTGTGTACTCCAAGAAGAGGAAGATGAGGCTAGACGTCTCTGCATGGAGTATgaga                     <  1:145774/291‑1 (MQ=255)`  
                                                                                                                                                                                                                                                                                   `aaCCTGATTGTATACAGTGACGGCTATTTATCACTAATGTCTACTTCCGGTCAATCCATCTACGTTAACCGAGGTGGCTATGTGTATGACCGAACGAGTCAATCAGACCGCTTTGACTCTGGTATTACGGTGAACATTATTCGTCTCCGCGACTACGATGAGATGCCTGAGTGCTTCCGTTACTGGATTGTCACCAAGGCTTCCCGTCAGTTCAACAACCGATTCTTTGGGGCACCGGAAGTAGAGGGTGTACTCCAAGAAGAGGAAGATGAGGCTAGACGTCTCTGCATGGAGTATgaga                     <  1:121030/301‑1 (MQ=255)`  
                                                                                                                                                                                                                                                                                    `aCCTGATTGTATACAGTGACAGCTATTTATCCCTAATGTCGACTTCCGGTCAATCCATCTACGTTAACCGAGGTGGCTATGTGTATGACCGAACGAGTCAATCAGACCGCTTTGACTCTGGTATTACTGTGAACATTATTCGTCTCCGCGACTACGATGAGATGCATGAGTGCTTCCGTTACTGGATTGTCACCAAGGCTTCCCGTCAGTTCAACAACCGATTCTTTGGGGCACCGGAAGTAGAGGGTGTACTCCAAGAAGAGGAAGATGAGGCTAGACGTCTCTGCATGGAGTATGAGAt                    <  1:229401/301‑1 (MQ=255)`  
                                                                                                                                                                                                                                                                                        `gATTGTATACAGTGACGGCTATTTATCCCTAATGTCTACTTCCGGTCAATCCATCTACGTTAACCGAGGTGGCTATGTGTATGACCGAACGAGTCAATCAGACCGCTTTGACTCTGGTATTACTGTGAACATTATTCGTCTCCGCGACTACGATGAGTTGCCTGAGTGCTTCCGTTACTGGATTGTCACCAAGGCTTCCAGTCAGTTCAACAACCGATTCTTTTGGGCACCGGAAGTAGAGGGTGTACTCCAAGAAGAGGAAGATGAGCCTAGACGTCTCTGCATGGAGTATGAGATGGAc                >  1:213281/1‑301 (MQ=255)`  
                                                                                                                                                                                                                                                                                        `gATTGTATACAGTGACGGCTATTTATCCCTAATGTCTACTTCCGGTCAATCCATCTACGTTAACCGAGGTGGCTATGTGTATGACCGAACGAGTCAATCAGACCGCTTTGACTCTGGTATTACTGTGAACATTATTCGTCTCCGCGACTACGATGAGATGCCTGAGTGCTTCCGTTACTGGATTGTCACCAAGGCTTCCCGTCAGTTCAACAACCGATTCTTTTGGGCACCGGAAGTAGAGGGTGTACTCCAAGAAGAGGAAGATGAGGCTAGACGTCTCTGCATGGAGTATGAGATGGag                >  1:92261/1‑300 (MQ=255)`  
                                                                                                                                                                                                                                                                                        `gATTGTATACAGTGACGGCTATTTATCCCTAATGTCTACTTCCGGTCAATCCATCTACGTTAACCGAGGTGGCTATGTGTATGACCGAACGAGTCAATCAGACCGCTTTGACTCTGGTATTACTGTGAACATTATTCGTCTCCGCGACTACGATGAGATGCCTGAGTGCTTCCGTTACTGGATTGTCACCAAGGCTTCCCGTCAGTTCAACAACCGATTCTTTGGGGCACCGGAAGTAGAGGGTGTACTCCAAGAAGAGGAAGATGAGGCTAGACGTCTCTGCATGGAGTATGAGATGGAc                >  1:250843/1‑301 (MQ=255)`  
                                                                                                                                                                                                                                                                                        `gATTGTATACAGTGACGGCTATTTATCCCTAATGTCTACTTCCGGTCAATCCATCTACGTTAACCGAGGTGGCTATGTGTATGACCGAACGAGTCAATCAGACCGCTTTGACTCTGGTATTACTGTGAACATTATTCGTCTCCGCGACTACGATGAGATGCCTGAGTGCTTCCGTTACTGGATTGTCACCAAGGCTTCCCGTCAGTTCAACAACCGATTCTTTGGGGCACCGGAAGTAGAGGGTGTACTCCAAGAAGAGGAAGATGAGGCTAGACGTATCTGCATGGAGTATGAGATGGaa                >  1:16499/1‑300 (MQ=255)`  
                                                                                                                                                                                                                                                                                        `gATTGTATACAGTGACGGCTATTTATCCCTAATGTCTACTTCCGGTCAATCCATCTACGTTAACCGAGGTGGCTATGTGTATGACCGAACGAGTCAATCAGACCGCTTTGACTCTGGTATTACTGTGAACATTATTCGTCTCCGCGACTACGATGAGATGCCTGAGTGCTTCCGTTACTGGATTGTCACCAAGGCTTCCCGTCAGTTCAACAACCGATTCTTTGGGGCACCGGAAGTAGAGGGTGTACTCCAAGAAGAGGAAGATGAGGATAGACGTCTCTGCATGGGGTATGAGATGGag                >  1:127597/1‑300 (MQ=255)`  
                                                                                                                                                                                                                                                                                        `cagtGTATACAGTGACGGCAAGTTATCCCTAATGTCTACTTCCGGTCAATCCATCTACGTTAACCGAGGTGGCTATGTGTATGACCGAACGAGTCAATCAGACCGCTTTGACTCTGGTATTACTGTGAACATTATTCGTCTCCGCGACTACGATGAGATGCCTGAGTGCTTCCGTTACTGGATTGTCACCAAGGCTTCCCGTCAGTTCAACAACCGATTCTTTGGGGCACCGGAAGTAGAGGGTGTACTCCAAGAAGAGGAAGATGAGGCTAGACGTCTCTGCATGGAGTATGAGATGGAc                <  1:226726/298‑1 (MQ=255)`  
                                                                                                                                                                                                                                                                                         `aTTGTATACAGTGACGGCTATTTATCCCTAATGTCTACTTCCGGTCAATCCATCTACGTTAACCGAGGTGGCTATGTGTATGACCGAACGAGTCAATCAGACCGCTTTGACTCTGGTATTACTGTGAACATTATTCGTCTCCGCGACTACGATGAGATGCCTGAGTGCTTCCGTTACTGGATTGTCACCAAGGCTTCCCGTCAGTTCAACAACCGATTCTTTGGGGCACCGGAAGTAGAGGGTGTACTCCAAGAAGAGGAAGATGAGGCTAGACGTCTCTGCATGGAGTATGAGATGGAcc               >  1:80967/1‑300 (MQ=255)`  
                                                                                                                                                                                                                                                                                         `aTTGTATACAGTGACGGCTATTTATCCATAATGTCTACTTCCGGTCAATCCATCTACGTTAACCGAGGTGGCTATGTGTATGACCGAACGAGTCAATCAGACCGCTTTGACTCTGGTATTACTGTGAACATTATTCGTCTCCGCGACTACGATGAGATGCCTGAGTGCTTCCGTTACTGGATTGTCACCAAGGCTTCCCGTCAGTTCAACAACCGATTCTTTGGGGCACCGGAAGTAGAGGGTGTACTCCAAGAAGAGGAAGATGAGGCTAGACGTCTCTGCATGGAGTATGAGATGGACt               >  1:115103/1‑301 (MQ=255)`  
                                                                                                                                                                                                                                                                                            `gTATACAGTGACGGCTATTTATCCCTAATGTCTACTTCCGGTCAATCCATCTACGTTAACCTAGGTGGCTATGTGTATGACCGAACGAGTCAATCAGACCGCTTTGACTCTGGTATTACTGTGAACATTATTCGTCTCCGCGACTACGATGAGATGCCTGAGTGCTTCCGTTACTGGATTGTCACCAAGGCTTCCCGTCAGTTCAACAACCGATTCTTTGGGGCACCGGAAGTAGAGGGTGTACTCCAAGAAGAGGAAGATGAGGCTAGACGTCTCTGCATGGAGTATGAGATGGACTACg            >  1:3198/1‑301 (MQ=255)`  
                                                                                                                                                                                                                                                                                            `gTATACAGTGACGGCTATTTATCCCTAATGTCTACTTCCGGTCAATCCATCTACGTTAACCGAGGTGGCTATGTGTATGACCGAACGAGTCAATCAGACCGCTTTGACTCTGGTATTACTGTGAACATTATTCGTCTCCGCGACTACGATGAGATGCCTGAGTGCTTCCGTTACTGGATTGTCACCAAGGCTTCCCGTCAGTTCAACAACCGATTCTTTTGGGCACCGGAAGTAGAGGGTGTACTCCAAGAAGAGGAAGATGAGGCTAGACGTCTCTGCATGGTGTATGAGATGGACTACg            >  1:194766/1‑301 (MQ=255)`  
                                                                                                                                                                                                                                                                                            `gTATACAGTGACGGCTATTTATCCCTAATGTCTACTTCCGGTCAATCCATCTACGTTAACCGAGGTGGCTATGTGTATGACCGAACGAGTCAATCAGACCGCTTTGACTCTGGTATTACTGTGAACATTATTCGTCTCCGCGACTACGATGAGATGCCTGAGTGCTTCCGTTACTGGATTGTCACCAAGGCTTCCCGTCAGTTCAACAACCGATTCTTTGGGGCACCGGAAGTAGAGGGTGTACTCCAATAAGAGGAAGATGAGGCTAGACGTCTCTGCATGGAGTCTgtctcttatacac            >  1:130468/1‑289 (MQ=255)`  
                                                                                                                                                                                                                                                                                              `ataCAGTGACGGCTATTTATCCCTAATGTCTACTTCCGGTCAATCCATCTACGTTAACCGAGATGGCTATGTGTATGACCGAACGAGTCAATCAGACCGCTTTGACTCTGGTATTACTGTGAACATTATTCGTCTCCGCGACTACGATGAGATGCCTGAGTGCTTCCGTTACTGGATTGTCACCAAGGCTTCCAGTCAGTTCAACAACCGATTCTTTGGGGCACCGGAAGTAGAGGGTGTACTCCAAGAAGAGGAAGATGAGGCTAGACGTCTCTGCATGGAGTATGAGATGGACTACGGt          <  1:310681/301‑1 (MQ=255)`  

                                                                                                                                                                                                                                                                                                         `|`                                                                                                                                                                                                                                                                                                           
`GGGGCCTCTAAACGGGTCTTGAGGGGTTTTTTGCTGAAAGGAGGAACTATATGCGCTCATACGATATGAACGTTGAGACTGCCGCTGAGTTATCAGCTGTGAACGACATTCTGGCGTCTATCGGTGAACCTCCGGTATCAACGCTGGAAGGTGACGCTAACGCAGATGCAGCGAACGCTCGGCGTATTCTCAACAAGATTAACCGACAGATTCAATCTCGTGGATGGACGTTCAACATTGAGGAAGGCATAACGCTACTACCTGATGTTTACTCCAACCTGATTGTATACAGTGACGACTATTTATCCCTAATGTCTACTTCCGGTCAATCCATCTACGTTAACCGAGGTGGCTATGTGTATGACCGAACGAGTCAATCAGACCGCTTTGACTCTGGTATTACTGTGAACATTATTCGTCTCCGCGACTACGATGAGATGCCTGAGTGCTTCCGTTACTGGATTGTCACCAAGGCTTCCCGTCAGTTCAACAACCGATTCTTTGGGGCACCGGAAGTAGAGGGTGTACTCCAAGAAGAGGAAGATGAGGCTAGACGTCTCTGCATGGAGTATGAGATGGACTACGGTGGGTACAA  >  NC_001604.1/24178‑24772`

  

Alignment Legend

Aligned base mismatch/match (shaded by quality score): `ATCG/ATCG` `< 3 ≤` `ATCG/ATCG` `< 11 ≤` `ATCG/ATCG` `< 25 ≤` `ATCG/ATCG` `< 36 ≤` `ATCG/ATCG` `< 38 ≤` `ATCG/ATCG`

Unaligned base: `atcg`    Masked matching base: `atcg`    Alignment gap: `‑`    Deleted base: `‑`
